# Intact Tails as a Welfare Indicator in Finishing Pigs? Scoring of Tail Lesions and Defining Intact Tails in Undocked Pigs at the Abattoir

**DOI:** 10.3389/fvets.2020.00405

**Published:** 2020-07-16

**Authors:** Anna Valros, Elina Välimäki, Heli Nordgren, Johannes Vugts, Emma Fàbrega, Mari Heinonen

**Affiliations:** ^1^Department of Production Animal Medicine and Research Centre for Animal Welfare, Faculty of Veterinary Medicine, University of Helsinki, Helsinki, Finland; ^2^Animal Sourcing, HKScan Finland Oyj, Forssa, Finland; ^3^Department of Veterinary Biosciences, Faculty of Veterinary Medicine, University of Helsinki, Helsinki, Finland; ^4^Animal Welfare Program, Institut de Recerca i Tecnologia Agroalimentària (IRTA), Monells, Spain

**Keywords:** tail biting, tail lesions, pig, meat inspection, abattoir, pig welfare

## Abstract

Tail biting lesions are a potential measure of on-farm animal welfare, as a large range of stressors increase the risk for tail biting outbreaks. Further, tail biting is a major challenge, as lesions due to tail biting decrease animal welfare and health, as well as production efficiency and carcass quality. The aim of this study was to suggest a tail scoring system for use at slaughterhouses processing undocked pigs, and to link tail lesion scores to meat inspection data. A further aim was to suggest a definition for an intact enough tail. To validate the suggested scoring system we assessed tails before and after scalding and compared results to pathological examinations. In total, 14,433 tails were scored, and 117 tails were collected for pathological examination. After scalding, 49.2% of all tails were scored as fully intact. Of tails with lesions 2.5% were scored as having major acute wounds (>2 cm), while 11.6% had minor acute wounds (<2 cm), and 36.7% healed lesions. Intact tails were on average 31.5 cm (SD 2.5 cm) long. Lesion scored at the slaughter-line agreed well with the pathological assessment. Tail lesions were associated with several meat inspection findings: tails with more severe lesions and of shorter length increased the risk for meat inspection findings to a higher degree. A detailed lesion scoring method helps to identify carcasses at risk for condemnations, as well as being a potential method for on-farm welfare estimation. We suggest that a system for scoring tail lesions in undocked pigs should utilize a combination of scoring of the lesion and measuring the tail length. As bite marks or bruises on an otherwise intact tail were not a concern for meat hygiene, we suggest the definition of an intact enough tail could allow the inclusion of tails with these mild changes. Meat inspection findings in carcasses with tails scored as healed, but with no fresh lesions, and with more than 75% of the average intact length remaining were rather similar to those of fully intact tails. Based on these findings we suggest that a tail of this length, and with no visible fresh lesions could also be considered intact enough.

## Introduction

Tail biting has a negative impact on the welfare of the pigs as well as the economics of production. Tail biting outbreaks cause an increase in production costs due to reduced animal performance, increased labor costs and higher healthcare costs (1–3). Further, tail biting lesions are known to be associated with an increased prevalence of carcass condemnations, due to especially abscesses and arthritis (4–6). In addition to causing problems *per se*, a high level of tail biting is considered a sign of a reduced welfare status on the farm (7), as tail biting is suggested to be an indication of increased stress experienced by the pigs (8, 9). Risk factors for this problem behavior include suboptimal housing, management, animal health, and feeding (10).

At the moment, there are several initiatives within the EU to enhance the enforcement of the EU ban on routine tail docking (11). In most countries within the EU the absolute majority of pigs are still docked (12). There is, however, no exact information on the proportion of docked pigs in different member states, and even less information on the prevalence of tail biting (13). Studies on tail lesions, both at farm, and at the slaughterhouse employ different types of scoring systems, which often makes comparison between studies difficult (10, 14). Also, on a more practical level, there has been a call for a harmonized scoring system of tail lesions:. The Pig welfare subgroup of the EU animal welfare platform proposed that to enhance the enforcement of the EU ban on tail docking, tail lesions should be assessed at the slaughterhouse, and that for benchmarking needs, the proportion of *intact* tails should be used (13). Using intact tails as a benchmark figure, instead of lesioned or docked tails, would allow a more constructive, positive discussion. However, interestingly enough, most studies do not define an intact tail any other way than that it lacks lesions [see e.g., (4–6, 15)]. In addition, the subgroup concluded that there is a need for collection of information on tail lesions of different type and severity, to be used in a feedback advisory system to farmers (13). There is, to our knowledge, no available definition for an intact tail, nor any general agreements for a system of classifying tail lesions at the slaughterhouse.

Many types of tail lesions have been reported: some are already healed by the time the pig reaches the slaughterhouse, some are fresh, and of different level of severity (4, 16). Further, especially in the case of non-docked pig populations, different proportions of tail lossare expected (16). For slaughterhouses to be able to focus on lesions with the highest relevance for carcass quality, it would be important to understand how tail lesions should be scored to identify those which pose a significant risk for meat inspection findings. It has been shown that the severity of the tail lesion is linked to an increase in the systemic inflammatory response of the pig (17). This is further supported by a recent study showing an increasing systemic temperature in pigs with tail lesions of increasing severity (18). Previous studies looking at the link between tail lesion severity and carcass condemnations have mainly included docked pigs [e.g., (5, 6, 19)]. Valros et al. (4) reported an increased risk of condemnations in carcasses with severely bitten tails in a population of mainly non-docked pigs. However, the scoring was not very detailed, and the proportion of docked pigs was not known. As far as we know, no studies exist looking at the link between proportion of tail loss, or remaining tail length, and carcass condemnations in detail. Tail docking has been totally forbidden in Finland since 2003, which allows for an evaluation of a potential lesion scoring system suitable for undocked pig populations.

The aim of this study was to improve the possibilities for animal-based welfare monitoring at the slaughterhouse by suggesting and validating a scoring system sensitive enough to properly differentiate farms with different levels of tail biting. The score is based on a tail lesion score and tail length, applicable for slaughterhouses and for undocked pigs. A central aim was to, together with the slaughterhouse, define an intact ***enough***tail—i.e., a tail which does not have substantial damage, and for which it is expected that the risk for meat inspection findings is not significantly increased. Secondary infections from tail lesions, potentially causing meat inspection findings, can reduce the welfare of affected pigs. How serious a tail lesion has to be for the biting *per se* to affect welfare, however, can only be assumed without live observations. To validate the suggested scoring system tails were assessed both before and after scalding, and slaughter-line scorings were compared to gross pathological examination and histopathological assessment of tails.

## Materials and Methods

### Data Collection at the Slaughterhouse

Data was collected at a large slaughterhouse in Finland during five consecutive days in June 2019. Producers sending their pigs for slaughter during this period were not notified of the study until after the data was collected. During these days, the vast majority of processed carcasses were scored, resulting in 14,433 scores. Using an earlier data from the same abattoir (4) it was, based on the prevalence of arthritis, calculated that 12,000 pigs should be enough to detect significant differences between pigs with intact vs. pigs with lesioned tails. Each carcass was available for scoring for a duration of ~7-8 s per scoring point (see below for more information on the scoring points A and B). All scoring and data recording were done by a team of eight persons, trained for the scoring before the data collection, using pictures and live observations. Scoring was further harmonized by live consultation between observers at the slaughter line whenever needed. Two pairs of two scorers were included in the core scoring team, being responsible for the quality of scoring, one pair per scoring point. At least one person from each core scoring team was present during all scoring sessions, and the additional members of the team only assisted the core scoring team members. During scoring, one person measured the length of each tail, as well as assessed the lesion, according to the scoring system described below. In total, two persons performed all scoring at point A and three persons at point B. Another person verified scoring when needed and recorded the observations.

#### Scoring Points

To get a comparison to the situation in the “live” pig, the first scoring point (point A) was situated just after bleeding, before the carcass had been processed in any way. The main scoring point (point B) was situated after singeing, whipping, bung drilling, and chest opening (further referred to as “after scalding”). At this point, the carcasses were hanged on gambrels, which allowed their individual identification throughout the rest of the slaughter process based on farm ID and running number.

As carcasses could change processing order on the line during the scalding process, and as they were only individually traceable after gambrelling, it was not possible to compare the scores between the two scoring points on an individual pig level. However, as the plant works on a schedule with line breaks about once an hour, it was possible to keep track of groups of pigs which were scored between each of these breaks (hereafter called “Sessions”) to allow for a comparison on group level of the data from the two scoring points. In total, 38 Sessions were scored during over the five observation days.

#### Tail Length Measurement and Scoring System

The tail length was measured at both scoring points (A and B) with a 50 cm long ruler with a 2 cm scale. The decision for the 2 cm measuring accuracy was based on a pilot visit, with the aim to get a reliable measurement at the rather high line speed. With the carcasses hanging upside down, the end of the ruler was placed on the dorsal side of the tail and firmly pushed toward the base of the tail. The tail was then manually extended against the ruler. Any hairs at the end of the tail were not included in the length measurement.

**Figure 1:**
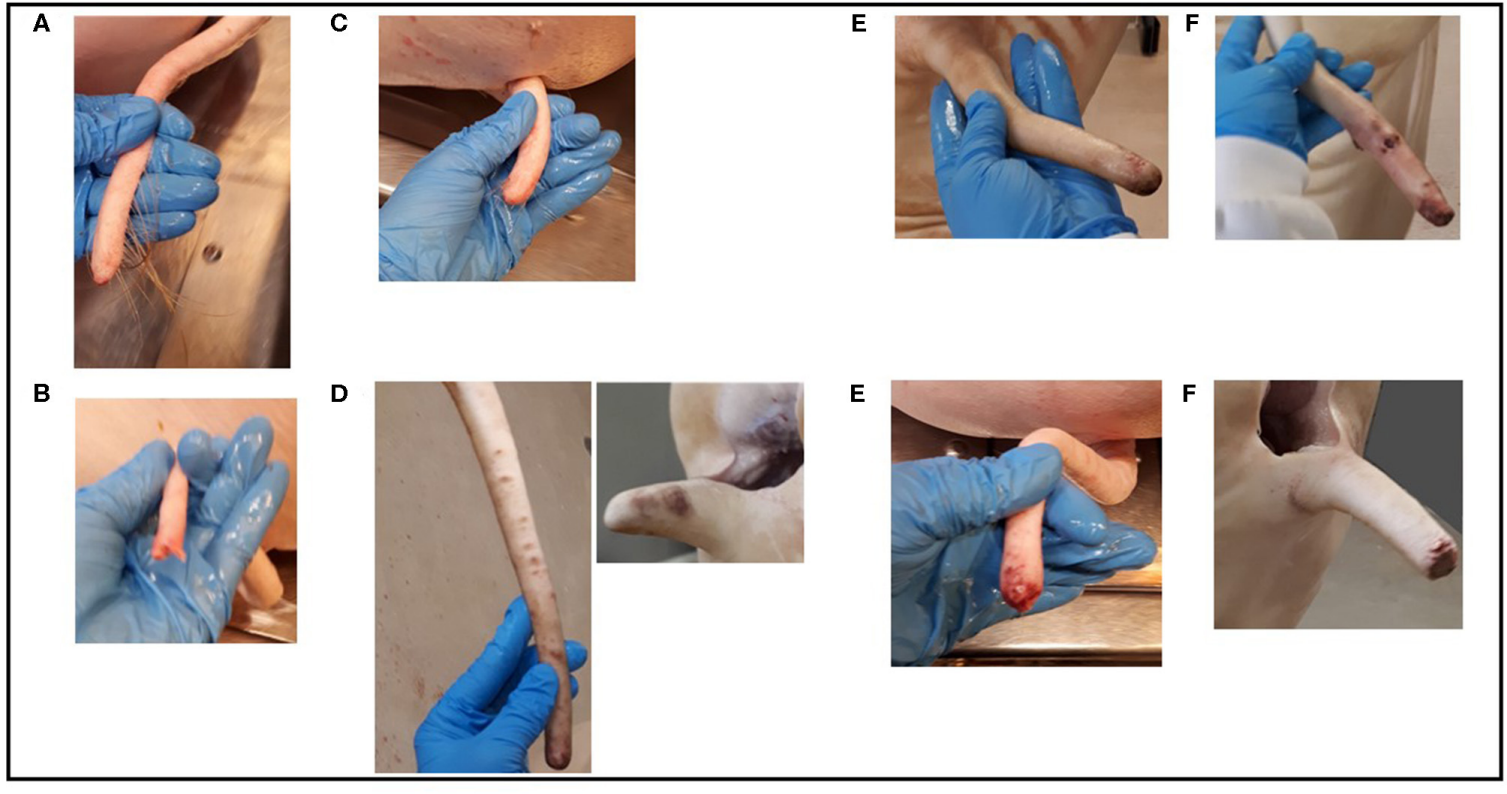
Example pictures of tails scored according to the scoring system used after scalding (See Table 1 and section Tail length measurement and scoring system for details): **(A)** intact tail; **(B)** broken tail; **(C)** healed tail; **(D)** healed tails with bite marks or bruises; **(E)** tails with minor acute wounds of different severity; **(F)** tails with major acute wounds of different severity. This figure was generated by Elina Välimäki.

Tail lesions were scored using the system described in Table 1 and Figure 1. Scoring was based on first visually inspecting, and then palpating the tail end for any changes in tissue texture or shape of the tail tip. Tails were also checked for lesions in other parts than the end. In long tails, the base part (~5 cm) of the tail was disregarded when scoring the category bite marks or bruises, as, bruises in this part were assumed to mainly be due to mechanical damage in the live pig, occurring e.g., due to crowded conditions during transport and ante mortem handling. The scoring system was developed as a combination of that developed by the FareWellDock-consortium (20) and that suggested by the EU pig subgroup (13). A preliminary scoring system was tested at the slaughter line during the pilot visit and amended accordingly. Due to the very different conditions, as well as differences in the appearance of the tail before and after scalding, the scoring scales had to be slightly different for each scoring point. At point A tails were sometimes dirty, hair was remaining and some tails had dry wound scabs, while at point B no dirt or wound scabs were left and almost all hair was gone, allowing for a more detailed inspection of the tail skin. If the tail had several lesions, the most severe one was scored. If the tail was considered healed, it was also checked and scored for potential acute damage. At point B intact and healed tails were also scored for bite marks or bruises (see Table 1 and Figure 1).

**Table 1:** Tail scoring system used at the slaughter line.

**Tail score**	**Definition, scoring system A**	**Definition, scoring system B**	**Combined scores used in statistical analyses, based on scoring system B**
Intact	The tail is fully intact, and long hairs grow out from the tail tip	The tail is fully intact, the end is rounded and slightly flattened	Intact Intact + Bite marks or bruises
Healed lesion	The tail is clearly shortened; the tail end is scarred, of abnormal shape or too thick to be intact. There may still be hairs at the end, but they do not grow from the entire tail tip	The tail is clearly shortened; the tail end is scarred, of abnormal shape or too thick to be intact. The skin is totally healed (no scab, wound or missing tissue).	Healed Healed + Bite marks or bruises
Acute lesion	There is fresh blood or a reddish scab on the tail, indicative of a lesion	There are either bite marks, bruises, or open wounds (missing skin tissue) on the tail	
Acute lesion: Bite marks or bruises^*^	NA*^1^	The tail has several small brown or red points, or long scratches, indicative of biting. These might be only bruises (no visible skin damage), or include minor skin damage, but with no tissue missing, and no visible wound; or the tail has a clear violet-colored bruising without tissue damage	
Acute lesion: Dry scab	There is a clear dry (brownish) scab/crust on the tail, usually at the end. No fresh (red) blood	NA*^1^	
Acute lesion: Minor wound	A wound with fresh blood or a reddish scab is present, clearly distinguishable from dirt or dry scab. Part of the tail might be missing. Wound is > 0, but <2 cm in diameter or length.	The tail has missing tissue, which has not fully healed yet; uneven “dents” in the skin; or a part of the tail is missing. Wound is > 0, but <2 cm in diameter or length.	Minor acute wound
Acute lesion: Major wound	A wound with fresh blood or a reddish scab is present, clearly distinguishable from dirt or dry scab. Part of the tail might be missing. Wound is 2 cm or larger in diameter or length	The tail has missing tissue, which has not fully healed yet; uneven “dents” in the skin; or a part of the tail is missing. Wound is 2 cm or larger in diameter or length	Major acute wound

*System A was used at scoring point A (after bleeding, before scalding) and system B at point B (after scalding)*.

* *Bite marks or bruises were not included in the overall tail score class Acute, as these were only scored on tails preliminarily scored as Intact or Healed;* *^1^ *NA, not applicable. In addition to the scores included in this table, some tails were additionally marked as “Broken” if they had lost their tip post mortem, during the scalding process*.

During scoring we also noted if the tails were swollen or not, but due to a high perceived uncertainty about the outcome, this parameter was not used in any analysis. Also Vom Brocke et al. (6) excluded swelling from their scoring key due to problems with a high inter-assessor disagreement. The reason mentioned in their study was confirmed by the current study: it is difficult to assess what a normal circumference of a tail should be. Further, even when palpating the tail, it was difficult to assess if the tissue texture was affected or not. During the pilot visit it was also noted that due to the carcass processing, a proportion of the tails had lost the tip by the time the tails were scored at point B. The tips, which could be found on the floor underneath the slaughter line where estimated to be ~2 cm. Thus, if tails were found to have a smooth and straight cut close to the tail end, they were first scored according to the scale in Table 1, but also given an additional remark of being “Broken.” The technical solutions differ between different slaughterhouses, thus this might not be a general problem. “Broken” was not added to the scoring system, as it was recorded to decrease the risk for errors at this specific site. In addition, some tail ends had a red-brownish color at point B due to the burning process. This discoloring was easily distinguishable from lesions, and was ignored at scoring.

#### Data From Meat Inspection

Data on meat inspection findings was made available by the slaughterhouse on an individual pig level. This data could be combined with the individual tail scores from point B based on the carcass ID number. The findings included in this study included: Whole carcass condemnations; Partial carcass condemnation; Abscesses, total; Abscesses in hip or back; Abscesses in other parts; Arthritis; Skin infection; Pericarditis; Pleuritis; Pneumonia and Organ condemnation. Even though some of the meat inspection findings were initially classified using a maximum of 4 scoring levels (0-3), all were transformed to binary variables (finding present or absent in the carcass) for analyses.

### Pathological Examinations

In order to validate the scoring performed at the slaughter line, a subset of tails was selected during one of the data collection days for pathological examinations. To get a representative sample, tails were selected to represent each type of tail lesions. In total, 117 tails were collected after they had been removed from the carcass, marked with their carcass ID and transported to the pathological laboratory during the same day. Eight tails were excluded from the pathological examination due to missing or indefinite identification, thus 109 tails were included in the study. Gross and histopathological results were combined, and the most severe lesion was used to give the final pathology score result to the tail.

#### Gross Pathology

The tails were palpated and inspected visually by a person blinded to the clinical scoring at the slaughterhouse. The tail was then cut transversally to allow inspection of the subcutaneous tissues, muscles and vertebra. Tails were recorded macroscopically as I = intact tail, B = broken post mortem during the slaughter process, H = healed tail (no visible granulation tissue formation) or C = chronic lesions where profound granulation tissue formation were detected and A = acute lesions including wounds, hemorrhages, necrosis, and acute inflammation.

#### Histopathology

The distal tail tip, ~3 cm in length and transected through the midsagittal plane, including skin, subcutaneous tissue, muscle and vertebra, was collected for the histopathological examination. If visible lesions continued more proximally, or lesions were macroscopically detected elsewhere in the tail, additional sampling was performed from the affected area. In these cases, a transfer section was cut from the first part of the tail where no visible pathological lesions were seen. The samples were fixed in 10% neutral buffered formalin and after fixation transferred into 14.28% EDTA (pH 7.0) for decalcification. The samples were processed routinely and stained with haematoxylin-eosin. Selected samples were further investigated with special stains (gram-stain for demonstrating the bacteria, Masson's trichrome staining for demonstrating fibrous tissue and immunohistochemical staining S-100 to visualize peripheral nerves).

Tails were classified in the histopathological examination as follows: 0 = no lesions, 1 = chronic lesions, including fibrous tissue cap around last vertebra and/or mild chronic dermal perivascular lympho-plasmasytic inflammation and/or neovascularization, 2 = mild superficial (epidermis and superficial dermis) neutrophilic inflammation, 3 = lesions extending to deep dermis and subcutis, and 4 = severe inflammations involving also muscle (myositis)/bone tissues (osteomyelitis) and abscess formation. If an acute inflammation was detected, the sagittal sample was investigated for possible spread of inflammation to the visible healthy part of tail and scored either present or absent.

The final pathology score was determined by combining information obtained from gross and histopathology examinations. If the histopathological examination revealed additional information to gross pathology scoring, such as an acute inflammatory process in tissue level not detected macroscopically the final score was determined based on the histopathology.

### Data Handling and Statistical Analyses

The data from scoring point A was only used as a comparison with the scores at point B on session level. All other analyses were based on data collected from individual pigs from point B.

The data was cleaned to exclude any clearly incorrect scores (such as tails scored as both healed and having an acute lesion, see Table 1; or tails scored as intact, but having a length measure below 10 cm). This left a data set of 14,419 tails scored at point A and 14,382 tails scored at point B (out of a total of 14,433 original scorings). The data from point B was further combined for analysis as indicated in the last column in Table 2. Further, to allow for estimation of the link between tail length and meat inspection findings in a practically applicable manner tails were classified according to length (point B only) into four (4) quarter-based classes: less than one-quarter of the full length left; one-quarter to one-half of the full length left; one-half to three-quarters of the full length left; and more than three-quarters of the full length left (21, 22). The average full tail length of intact tails in the current study (see results section) was used as the reference value for expected total tail length, resulting in the following tail length classes: <9 cm; 9–16 cm; 17–24 cm; >24 cm.

**Table 2:** Percentage (and number) of tail scores at scoring points A (after bleeding, before scalding) and B (after scalding) in finishing pigs at the slaughter line.

	**Scoring point A, % (*n*)**	**Scoring point B, % (*n*)**
Tail lesion score		
Intact, total^*^	58.7% (8471)	49.2% (7080)
Intact + Bite marks or bruises^*^^1^	NA^*^^3^	*11.4% (1639)*
Healed lesion, total	22.6% (3264)	36.7% (5281)
*Healed lesion + Bite marks or bruises*	NA^*^^3^	*9.1% (1309)*
Acute, total *(not including Bite marks or bruises)*	18.7% (2694)	14.1% (2021)
Dry scab ^*^^2^	*9.2% (1329)*	NA^*^^3^
*Minor wound (>0 to <2 cm)*	*6.5% (942)*	*11.6% (1664)*
*Major wound (≥2 cm)*	*2.8% (407)*	*2.5% (357)*
Tail length class		
> 24 cm		81.7% (11 756)
17-24 cm		15.0% (2152)
9-16 cm		2.6% (379)
< 9 cm		0.06% (94)

*Scores in italics are subsets of the overall tail score classes (Intact, Healed, Acute)*.

* *Includes Broken tails (point B): the tip of the tail lost due to carcass processing*.

*1 *Bite marks or bruises were only scored at point B*.

*2 *Dry scabs were only scored at point A*.

*3 *NA, not applicable*.

All statistical analyses were performed using IBM SPSS statistics version 25.

To assess if Broken tails with no signs of lesions could be considered as Intact, a preliminary analysis was performed, where the level of meat inspection findings in Intact and Broken tails was compared to tails scored as Intact, but not Broken, using Chi-squared analysis.

To compare results between the two scoring points (A and B), the percentage of tails within each of the overall tail score classes (Intact, Healed and Acute) was calculated for each session (*n* = 38) and for both scoring points (A and B). Pearson's correlations were then applied to test how well the two scoring points agreed on this overall scoring. The same procedure was further performed for tail length: average tail length was calculated for each session for scoring point A and B separately, and then the two scoring points were compared with Pearson's correlations. As the scoring for the severity of acute lesions differed between points A and B and as Bite marks and bruises were only scored at point B, comparisons were only done for overall tail score classes (Intact, Healed, and Acute).

The association between different tail lesion scores and tail length classes and meat inspection findings was tested using logistic regression models for each of the findings separately. As tail lesion score and tail length class were highly correlated (based on an initial Chi-square test, results not reported), separate models were run for each variable. Different types of tail lesions were always compared to intact tails, and the shorter tail length classes to tails > 24 cm. Finally, to further assess if tail lesions were associated with meat inspection findings also when only a minor part of the tail was missing (tail > 24 cm) models for each meat inspection finding were performed with tail score as the categorical variable separately for data including only tails of the longest tail length class (>24 cm). The goodness-of-fit of each model was ensured using the Hosmer & Lemeshow test.

In those carcasses where meat was condemned possible differences in amount (kg) of condemned meat between carcasses with different tail lesion scores and of different lengths were tested using Kruskal-Wallis tests, followed by Bonferroni-corrected pairwise comparisons when appropriate.

## Results

### Broken Tail Ends

In total, 3,471 tails were scored as Intact but Broken at point B, while another 3,625 Intact tails were scored as not Broken. Broken tails were on average 28.9 cm (SD 2.7) long, i.e., 2.6 cm shorter than Intact tails (see below). For the prevalence of most meat inspection findings, there was no difference between Intact tails with Broken ends and those scored as not Broken (*p* > 0.10 for all findings except for arthritis and pleuritis and partial carcass as well as organ condemnations). The occurrence of arthritis (Pearson Chi^2^ = 6.0, *p* = 0.01), pleuritis (Pearson Chi^2^ = 30.6, *p* < 0.001), partial carcass condemnations (Pearson Chi^2^ = 8.8, *p* = 0.003) and organ condemnations (Pearson Chi^2^ = 6.4, *p* = 0.01), however, was actually higher in carcasses with tails that were not Broken, as compared to in those carcasses with Broken tails. As this raised no concern that Broken tails would not have been Intact, we decided to accept the broken tails as part of the category Intact. This decision was further supported by the results of the pathological examinations (reported below): none of the tails scored Broken (*n* = 9) at the slaughterhouse were found to have lesions.

### Descriptive Data

**Figure 2:**
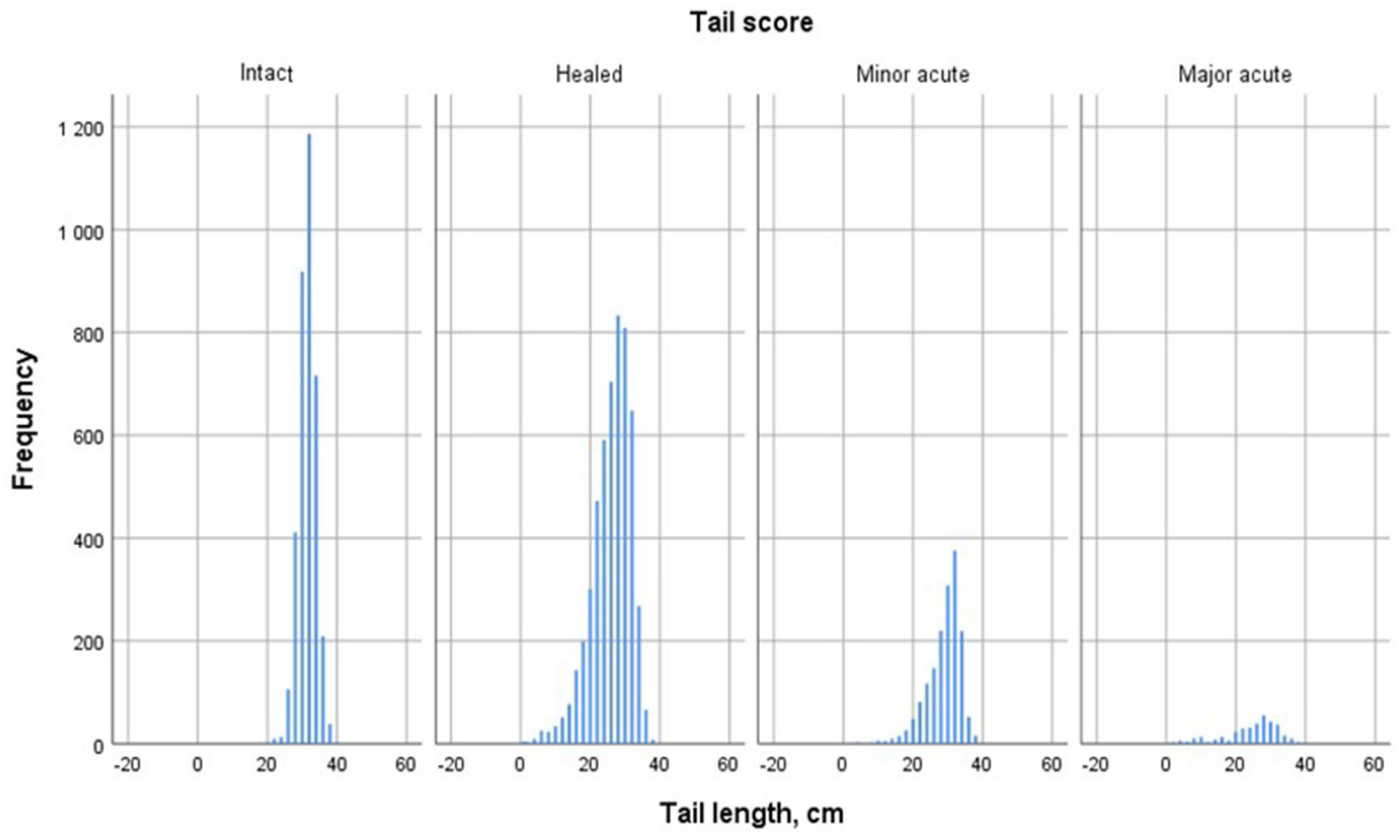
Distribution of tail lengths in finishing pigs with different lesion scores, measured after the entire scalding process at the slaughter line (*n* = 10 889).

The distribution of tail length by tail lesion score at point B, is shown in Figure 2, not including Broken tails in the Intact tail data. The average tail lengths at point B was 31.5 cm (SD 2.5) for Intact tails, excluding Broken tails (*n* = 3612), 26.1 cm (SD 6.2) for tails with Healed lesions (*n* = 5271), 28.9 cm (SD 5.0) for tails with Minor acute lesions (*n* = 1653) and 24.5 cm (SD 7.8) for tails with Major acute lesions (*n* = 353). For comparison, at point A, Intact tails (*n* = 8 465, including also tail later scored Broken) were measured to be 31.6 cm (SD 2.8). Descriptive data of tail lesion scorings from points A and B, and tail length classes are presented in Table 2.

### Validation of the Scoring System

#### Pathology

The scoring performed at the slaughterhouse and the results of the pathological examination were mostly converging: In 90% of tails (98/109) the results agreed. In one tail, recorded as an intact tail at the slaughterhouse, a mild superficial inflammation (score 2) was detected in the histopathological examination. Five tails scored as having healed lesions at the slaughterhouse were found to have acute lesions according to the histopathology: In four of these an abscess formation (score 4) was detected and in one a mild superficial inflammation (score 2). The remaining six tails with a difference in results were recorded at the slaughterhouse as having acute lesions, but the histopathological examination revealed only mild chronic lesions. It is possible however, that some superficial inflammatory changes were no longer visible in the histopathological examination, due to the dehairing measures (burning) affecting the quality of histopathological samples, especially on superficial parts (epidermis and superficial dermis). A comparison between results from slaughterhouse and pathology results (gross and histopathology combined) is presented in the Table 3.

**Table 3:** Comparison between the tail scoring results of finishing pigs obtained at the slaughterhouse and during pathological evaluation (*n* = 109).

		**Pathology results**				
		**Intact**	**Broken**	**Acute**	**Chronic/Healed**	**Total, *n***
Slaughterhouse scoring	Intact	95% (19)	0% (0)	5% (1)	0 (0)	20
	Broken	0% (0)	100% (9)	0% (0)	0% (0)	9
	Acute	0% (0)	0% (0)	89% (33)	11% (4)	37
	Healed	0% (0)	0% (0)	12% (5)	88% (38)	43

*The cells indicate within-row-percentages (and number of tails), namely number of slaughterhouse scorings within each class of the pathology scoring*.

#### Comparison Between Results From Scoring Point a and Point b

Each session (*n* = 38) contained between 353 and 605 pigs (average 378). The percentage of different types of tail scores per session for each of the scoring points A and B correlated moderately for Intact tails (*r*_p_ = 0.32, *p* = 0.048) and for Healed tails (*r*_p_ = 0.51, *p* = 0.001) but not for Acute tails (*r*_p_ = 0.21, *p* = 0.2). The mean tail length for the Sessions, as measured at point A and B correlated moderately (*r*_p_ = 0.38, *p* = 0.02).

### Associations Between Tail Lesions and Tail Length, and Meat Inspection Findings

Higher levels of both whole and partial carcass condemnations were found in carcasses with tails with most lesion types, and in shorter tails (Table 4A). Most meat inspection findings were also more commonly recorded in carcasses with lesioned tails as compared to intact ones, and in tails shorter than 24 cm (Tables 4A,B). The only type of lesion which was not associated with carcass condemnations was an intact tail with bite marks or bruises. This type of lesion was, however, associated with a higher level of skin infections (Table 4B). There was no association between either tail lesion score or tail length class and organ condemnations (*p* > 0.1 for both).

**Table 4A:** Results from logistic regressions on the association between (a) tail lesion score and (b) tail length class (separate models) and carcass condemnations as well as abscesses recorded at meat inspection (*n* = 14,382).

	**Whole carcass condemnation**	**Partial carcass condemnation**	**Abscesses, total**	**Abscesses in hip or back**	**Abscesses in other parts**
	%^*^^1^, OR^*^^2^ (CI)^*^^3^	**%, OR (CI)**	**%, OR (CI)**	**%, OR (CI)**	**%, OR (CI)**
(a) Tail lesion score^a^	0.1 ^***^	4.0 ^***^	2.3 ^***^	0.4 ^***^	1.4 ^***^
Intact tail + bite marks or bruises	0.1, 0.66 (0.08-5.68)^ns^	4.6, 1.18 (0.90-1.54)^ns^	2.1, 0.92 (0.63-1.34)^ns^	0.2, 0.43 (0.13-1.44)^ns^	1.5, 1.05 (0.66-1.67)^ns^
Healed lesion	0.3, 2.74 (0.94-8.03)^†^	5.8, 1.34 (1.22-1.80)^***^	3.4, 1.48 (1.16-1.90)^**^	1.4, 3.23 (1.99-5.30)^***^	1.5, 1.05 (0.74-1.48)^ns^
Healed lesion + bite marks or bruises	0.2, 2.48 (0.57-10.4)^ns^	6.6, 1.72 (1.33-2.23)^***^	4.4, 1.96 (1.42-2.69)^***^	1.7, 4.03 (2.24-7.35)^***^	2.6, 1.88 (1.25-2.83)^**^
Minor acute wound	0.4, 4.59 (1.46-14.5)^**^	7.2, 1.86 (1.48-2.35)^***^	4.7, 2.10 (1.58-2.80)^***^	1.9, 4.62 (2.70-7.91)^***^	2.5, 1.78 (1.22-2.62)^**^
Major acute wound	2.8, 31.3 (10.6-92.2)^***^	20, 6.11 (4.56-8.18)^***^	17, 8.35 (6.00-11.6)^***^	5.6, 14.0 (7.60-25.7)^***^	6.2, 4.64 (2.85-7.55)^***^
(b) Tail length class^b^	0.1 ^***^	4.7 ^***^	2.7 ^***^	0.6 ^***^	1.6 ^**^
17-24 cm	0.6, 4.11 (1.94-8.71)^***^	8.3, 1.81 (1.52-2.16)^***^	5.5, 2.11 (1.70-2.62)^***^	2.5, 4.49 (3.13-6.45)^***^	2.4, 1.52 (1.11-2.07)^**^
9-16 cm	1.1, 7.83 (2.60-23.5)^***^	9.8, 2.17 (1.53-3.08)^***^	7.9, 3.10 (2.10-4.58)^***^	5.0, 9.21 (5.47-15.5)^***^	2.6, 1.66 (0.87-3.16)^ns^
< 9 cm	4.3, 32.6 (10.7-99.5)^***^	29, 8.09 (5.13-12.7)^***^	28, 13.8 (8.67-22.0)^***^	15, 30.5 (16.5-56.6)^***^	4.3, 2.72 (0.99-7.48)^†^

*1 *% of findings within tail lesion score or length category*,

*2 Odds ratio,

*3 95% confidence interval for OR,

a *Intact tails act as the reference category, significance level given is for the entire model in this row*,

b *Tails length class 4 (>24 cm) acts as the reference category*.

ns p > 0.1;

† p < 0.1;

*p < 0.05;

** p < 0.01;

*** *p < 0.001*.

**Table 4B:** Results from logistic regressions on the association between (a) tail lesion score and (b) tail length class (separate models) and arthritis, pericarditis, pleuritis, pneumonia, and skin infection recorded at meat inspection (*n* = 14 382).

	**Athritis**	**Pericarditis**	**Pleuritis**	**Pneumonia**	**Skin infection**
	%^*^^1^, OR^*^^2^ (CI)^*^^3^	**%, OR (CI)**	**%, OR (CI)**	**%, OR (CI)**	**%, OR (CI)**
(a) Tail lesion score^a^	1.3^***^	5.0^***^	39^**^	2.3^***^	0.4^***^
Intact tail + bite marks or bruises	1.4, 1.05 (0.65-1.68)^ns^	5.4, 1.09 (0.85-1.39)^ns^	39, 0.99 (0.88-1.11)^ns^	2.9, 1.24 (0.88-1.73)^ns^	1.4, 3.67 (2.03-6.65)^***^
Healed lesion	2.4, 1.80 (1.32-2.45)^***^	7.2, 1.48 (1.25-1.76)^***^	42, 1.10 (1.02-1.20)^*^	2.8, 1.23 (0.95-1.58)^ns^	0.3, 0.85 (0.42-1.69)^ns^
Healed lesion + bite marks or bruises	2.1, 1.55 (0.99-2.42)^†^	6.6, 1.36 (1.06-1.75)^*^	44, 1.19 (1.05-1.35)^**^	2.2, 0.95 (0.63-1.43)^ns^	0.7, 1.77 (0.82-3.91)^ns^
Minor acute wound	2.0, 1.53 (1.02-2.31)^*^	6.3, 1.29 (1.02-1.63)^*^	42, 1.11 (0.99-1.24)^†^	3.1, 1.35 (0.97-1.87)^†^	0.8, 2.03 (1.02-4.07)^*^
Major acute wound	8.4, 6.75 (4.35-10.5)^***^	7.3, 1.50 (0.99-2.28)^†^	36, 0.89 (0.71-1.11)^ns^	12, 5.58 (3.86-8.01)^***^	2.0, 5.16 (2.18-12.2)^***^
(b) Tail length class^b^	1.6^***^	5.6^***^	40^**^	2.6^**^	0.6^***^
17-24 cm	3.2, 2.00 (1.51.2.64)^***^	7.5, 1.37 (1.14-1.63)^**^	43, 1.09 (0.98-1.20)^†^	3.8, 1.45 (1.13-1.87)^**^	0.5, 0.80 (0.41-1.56)^ns^
9-16 cm	4.0, 2.48 (1.45-4.24)^**^	10, 1.88 (1.33-2.66)^***^	36, 0.83 (0.67-1.02)^ns^	3.7, 1.43 (0.83-2.46)^ns^	0.8, 1.37 (0.43-4.38)^**^
<9 cm	6.4, 4.11 (1.77-9.50)^**^	5.3, 0.95 (0.38-2.34)^ns^	28, 0.57 (0.36-0.89)^*^	7.4, 2.99 (1.37-6.51)^**^	5.3, 9.66 (3.80-24.5)^***^

*1 % of findings within tail lesion score or length category,

*2 Odds ratio,

*3 95% confidence interval for OR,

a Intact tails act as the reference category, significance level given is for the entire model in this row,

b Tails length class 4 (> 24 cm) acts as the reference category.

ns p > 0.1;

† p < 0.1;

* p < 0.05;

** p < 0.01;

*** *p < 0.001*.

When only carcasses with tails of the longest tail length class (>24 cm) were included in the analysis, the association between tail lesion scores and most meat inspection findings, as well as partial carcass condemnations, remained significant (Tables 5A,B). Whole carcass condemnations however, were not found to be associated with tail lesion score in this tail length class, and for pleuritis the association was only a tendency (*p* = 0.08). Carcasses with intact tails, and bite marks or bruises, had a very similar level of meat inspection findings as those with fully intact tails—the only association was to skin infections (Table 5B). In carcasses with healed tail lesions, and no additional bite marks or bruises, there was only a significant association to pericarditis (Table 5B). Healed lesions, in combination with bite marks or bruises were associated with partial carcass condemnations, abscesses (Table 5A), and pleuritis (Table 5B). Further, both minor and major acute wounds were associated with an increase in partial carcass condemnations, and major acute wounds were additionally associated with almost all meat inspection findings.

**Table 5A:** Results from logistic regressions on the association between tail lesion score, when the tail was at least 24 cm (*n* = 11756) and carcass condemnations as well as abscesses recorded at meat inspection.

	**Partial carcass condemnation**	**Abscesses, total**	**Abscesses in hip or back**	**Abscesses in other parts**
	%^*^^1^, OR^*^^2^ (CI)^*^^3^	**OR (CI)**	**OR (CI)**	**OR (CI)**
Tail lesion score^a^	3.9^***^	2.3^***^	0.4^***^	1.4^***^
Intact tail + bite marks or bruises	4.6, 1.19 (0.90-1.56)^ns^	2.1, 0.90 (0.61-1.33)^ns^	0.1, 0.85 (0.07-1.28)^ns^	1.5, 1.05 (0.65-1.68)^ns^
Healed lesion	4.9, 1.19 (1.00-1.58)^†^	2.7, 1.18 (0.87-1.59)^ns^	0.6, 1.54 (0.81-2.94)^ns^	1.3, 0.96 (0.63-1.45)^ns^
Healed lesion + bite marks or bruises	5.6, 1.46 (1.06-2.02)^*^	4.0, 1.76 (1.20-2.60)^**^	1.2, 2.82 (1.33-5.98)^**^	2.9, 2.14 (1.35-3.40)^**^
Minor acute wound	5.9, 1.53 (1.17-2.00)^**^	3.3, 1.44 (1.01-2.04)^†^	0.7, 1.78 (0.84-3.77)^ns^	2.1, 1.51 (0.97-2.34)^†^
Major acute wound	15, 4.36 (2.90-6.54)^***^	8.7, 4.74 (2.43-6.83)^***^	3.4, 8.39 (3.54-19.9)^***^	3.4, 2.50 (1.14-5.51)^*^

*1 % of findings within tail lesion score category,

*2 Odds ratio,

*3 95% confidence interval for OR,

a *Intact tails act as the reference category, significance level given is for the entire model in this row*.

ns p > 0.1;

† p < 0.1;

* p < 0.05;

** p < 0.01;

*** *p < 0.001*.

**Table 5B:** Results from logistic regressions on the association between tail lesion score, when the tail was at least 24 cm (*n* = 11756) and arthritis, pericarditis, pleuritis, pneumonia, and skin infection recorded at meat inspection.

	**Athritis**	**Pericarditis**	**Pleuritis**	**Pneumonia**	**Skin infection**
	%^*^^1^, OR^*^^2^ (CI)^*^^3^	**OR (CI)**	**OR (CI)**	**OR (CI)**	**OR (CI)**
Tail lesion score^a^	1.4^**^	5.0^*^	39 ^†^	2.4^***^	0.4^***^
Intact tail + bite marks or bruises	1.5, 1.06 (0.66-1.71)^ns^	5.4, 1.09 (0.85-1.40)^ns^	39, 1.01 (0.90-1.13)^ns^	2.9, 1.23 (0.87-1.73)^ns^	1.4, 3.89 (2.10-7.20)^***^
Healed lesion	1.9, 1.42 (0.98-2.05)^†^	6.3, 1.30 (1.06-1.60)^*^	42, 1.10 (1.00-1.21)^†^	2.5, 1.07 (0.79-1.45)^ns^	0.4, 1.00 (0.45-2.20)^ns^
Healed lesion + bite marks or bruises	1.6, 1.20 (0.68-2.14)^ns^	6.5, 1.35 (1.00-1.81)^†^	44, 1.21 (1.04-1.40)^*^	2.1, 0.89 (0.54-1.46)^ns^	0.5, 1.30 (0.44-3.83)^ns^
Minor acute wound	1.8, 1.31 (0.82-2.08)^ns^	5.9, 1.21 (0.94-1.57)^ns^	41, 1.10 (0.97-1.24)^ns^	2.6, 1.10 (0.75-1.60)^ns^	0.7, 2.06 (0.96-4.45)^†^
Major acute wound	5.3, 4.07 (2.13-7.80)^***^	8.3, 1.73 (1.04-2.88)^*^	41, 1.09 (0.82-1.45)^ns^	10, 4.67 (2.88-7.59)^***^	1.9, 5.47 (1.85-16.2)^**^

*1 *% of findings within tail lesion score category*,

*2 *Odds ratio*,

*3 *95% confidence interval for OR*,

a *Intact tails act as the reference category*,

*^b^Tails length class 4 (> 24 cm) act as the reference category*.

ns p > 0.1;

† p < 0.1;

* p < 0.05;

** p < 0.01;

*** *p < 0.001*.

The amount of meat condemned per carcass (excluding carcasses with zero condemnation) did not differ between carcasses with different tail lesion scores (Kruskall-Wallis Chi^2^ = 8.56, *df* : 5, *p* > 0.1) but differed between different tail length classes (Chi^2^ = 20.18, *df* = 3, *p* < 0.001). Carcasses with more than half of the tail missing had a higher amount of meat condemned [ <9 cm (*n* = 31): median 3.30 kg (interquartile range 8.70); 9-16 cm (*n* = 41): 3.80 kg (5.35)] than carcasses with tails over 24 cm [*n* = 588, 2.40 kg (2.86)] (*p* < 0.05 for both pairwise comparisons). Carcasses with tails in the length class 17-24 cm (*n* = 191) were intermediate and tended to have more meat condemned [2.50 kg (2.23)] than carcasses with tails > 24 cm (*p* < 0.1).

## Discussion

The results are promising for developing a measure based on intact tails to estimate on-farm pig welfare. Further, we show that it is possible to perform a detailed enough tail scoring to identify different types of lesions on a large-scale slaughterhouse line. Thus, lesions, which correspond to different levels of meat inspection findings, can be separated. Recording both tail length and lesion severity gives additional information compared to recording only one of these. The results further indicate that tails, which are otherwise intact, but have mild lesions, defined as bite marks or bruises might not be a significant risk for meat hygiene.

### Scoring System

**Figure 3:**
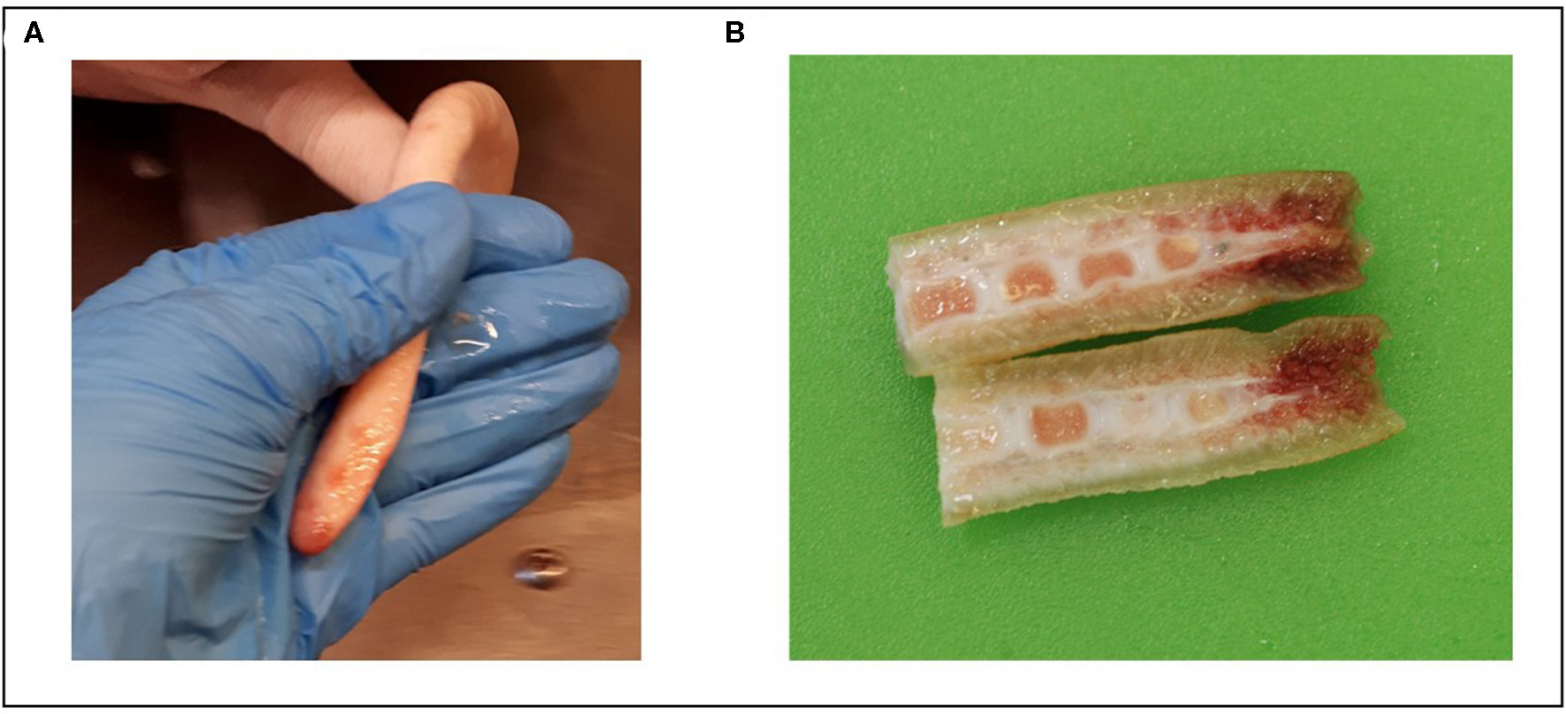
Picture **(A)** shows a fully intact tail at the slaughter line, showing the flattening tail end, and picture **(B)** a transversally cut tail end with a lesioned end, but where the flattening shape of the last vertebrae is visible. Panel **(A)** was generated by Elina Välimäki and Panel **(B)** was generated by Heli Nordgren.

The chosen scoring system was developed to be as detailed as possible, while also being feasible for application at the slaughter line. Some considerations did come up during the data collection. Firstly, the limits for minor and major acute wounds was based on a preliminary recommendation by the EU animal welfare platform Pig welfare subgroup (based on a document by Keeling and Valros, 2019, *unpublished*) for scoring of tails at the slaughterhouse. However, the 2 cm limit was rather arbitrary and might be too high: when collecting the current data, it was observed that most minor wounds were actually only a few millimeters. Secondly, the definition of intact tails, before scalding, as having long hairs growing out from the tail tip was found to be non-reliable, as many tails of clearly shortened length, and with clearly scarred tissue at the tail tip still had long hairs covering the entire tail end. Hairs could also be seen after scalding in some cases (see Figure 1C). Pathological examination showed that an intact tail can be identified by the presence of the full last vertebrae, which can be identified by its flattening shape also by palpating the tail (see Figure 3). However, the distinction of fully intact and healed tails often required palpation of the tail end, thus not being very plausible in a practical situation, and not possible if an automatic camera-based system [such as those proposed by Larsen et al. (23) and Blömke et al. (24)] was to be employed. Finally, the issue with the broken tail ends shows that it is important to adjust any scoring system to the practical situation of a slaughterhouse.

The scoring performed before and after scalding did not give exactly the same result and were only moderately correlated. However, the level of intact tails was rather comparable, especially as compared to the study by Carroll et al. (25), where a much larger discrepancy between two similar scoring points was reported in docked pigs. One reason for it being difficult to score tails in a similar way before and after scalding is that the appearance of the tail is very different. Before scalding tails were still covered with hair, and sometimes very dirty. Scabs covering the end of the tail also made it difficult to assess the size or acuteness of the underlying lesion. Scoring before scalding probably gives a better estimate of what the producer him/herself might have seen, while the scoring after scalding is more accurate, as also observed by Carroll et al. (25).

When comparing scores given at the slaughter line with those resulting from the much more detailed pathological exam, it appears that the scoring system used in the current system was indeed rather reliable. It needs to be noted, however, that especially dehairing the tails by burning affected the quality of the histopathological samples to some degree. As this mainly concerned superficial parts of the tail, it is possible that some clinically observed acute, but minor changes were no longer visible in the histopathological examination. The result is surprisingly positive, as previous experience has shown that at least in live pigs, it is difficult to assess tail damage merely by clinical examination (26). This further supports the suggestion that it might be more reliable to score tails after the scalding process than before.

The tail length measuring was, for practical reasons, only done to an accuracy of 2 cm. However, the result does seem to be in reasonable agreement with the study by Herskin et al. (22), which reported an average tail length of 30.6 cm in Danish Landrace x Yorkshire x Duroc pigs As far as we know, no information is available on the effect of pig breed or age on tail length, thus this result can so far only be applied to the specific Finnish pig population. The vast majority of the pigs in this study were crosses between Norwegian Landrace x Topigs York Z sows and Danish Duroc sires and the average carcass weight of the pigs was 89.7 kg (SD 7.38 kg).

### Tail Lesions and Length, Meat Condemnations and Meat Inspection Findings

The results of a significant association between tail lesions and the different meat inspection findings, as well as carcass condemnations is not novel, nor surprising. Similar results have been shown in several previous studies [see e.g., (4–6)]. The separation between healed and fresh lesions in this study, however, provides some novel insights. We show that healed lesions are also linked to an increase in some meat inspection findings. As far as we are aware, previous studies do not differentiate healed lesions, but include these in, for example, the mild lesion category [e.g., (15, 16)]. However, even when tails appear healed, there might still be underlying ongoing infections: in our study, four tails recorded as healed in the slaughterhouse, as well as during the gross pathology scoring, proved to have deep abscesses in the histopathological examination.

To the best of our knowledge, this is the first study to look at an association between tail length and carcass condemnations or meat inspection findings. It was clear that with a larger part of the tail missing, i.e., a shorter remaining tail length, the higher was the level of secondary infections. As a consequence, the amount of condemned meat was also higher in carcasses with a large proportion of the tail missing. This could be due to several reasons. The tail might have been bitten repeatedly, thus providing several time points for infections. It might also be that even if a tail is bitten off in one incidence, which could be possible e.g., if it was a case of sudden-forceful biting (27), a larger wound, cutting through more tissue could increase the risk of infections due to a prolonged healing period. Also, a larger wound provides a larger area for contamination. Finally, very little is known about the anatomy of pig tails, but it might be possible, that there are differences in the different parts of the tail that affect the spread of infections. It has been shown by Herskin et al. (28) that the behavioral reaction of pigs to tail docking is larger, the larger a proportion of the tail is amputated, which does indicate that there might be differences in the anatomical characteristics of different parts of the tail.

### What Is an Intact Enough Tail?

The data shows that neither the risk for most meat inspection findings (except the skin infection), nor for carcass condemnations, was increased if there were mere bite marks or bruises on an otherwise intact tail. This indicates that these minor lesions are not important for meat hygiene, and probably also of only minor relevance for pig welfare. On the other hand, even in tails which were above 75% of the average length of an intact tail (over 24 cm), acute lesions, and especially major ones, caused a significant risk for increased condemnations and meat inspection findings. It is thus very important to avoid all tail lesions, even when these do not escalate to the point where a large proportion of the tail is lost. However, it is especially crucial not to allow tail biting to continue when wounds have first appeared: major acute wounds did seem to pose a serious risk for meat hygiene, even when 75% or more of the tail remained.

As healed tails, especially when reasonably long, were difficult to separate from intact tails, it would have been convenient, from a practical point-of-view if there had been no association between healed tails and meat hygiene in these long tails. This was (almost) the case for healed tails with no further bruises—for these there was only a tendency for an increased level of partial carcass condemnations, as well as a significant association to pericarditis. However, if the healed tails were also recorded to have bite marks or bruises, this was not so clear: for example, the prevalence of abscesses (4.0%) was actually higher than in the case of tails with minor acute wounds (3.3%).

In practice, scoring of an intact enough tail will always be a compromise, as fully intact is a matter of definition, unless at least a physical palpation is performed, and as tail length varies (between 18 and 42 cm for intact tails in this study). Based on the findings of this study we suggest that an intact enough tail could be defined as a tail of at least 75% of the average fully intact tail length in the specific population, and with no signs of any kind of biting lesions. Of the pigs included in this study, only 0.7% had intact tails below 24 cm of length (i.e., below 75% of the average full tail length of intact tails). Thus, even though intact tails can vary in length, setting the limit at 75% would not have resulted in a major misclassification of intact tails into lesioned ones. The length limit is further supported by the results from the study by Herskin et al. (28), which indicated a lower pain reaction at docking, and thus less of a negative welfare impact, when 25% of the tail was amputated, compared to larger proportions. For practical reasons, however, it might not be possible to identify bite marks or bruises reliably at the slaughter line. For example, the automatic tail lesion detection system, TailCam, developed in the PigWatch project (23) does not consider superficial scratches as related to tail biting, and even the accuracy of separating small lesions from no lesions is not yet fully convincing. We thus suggest that an intact enough tail for this population of pigs could be defined as a tail over 24 cm long and having no acute wounds. If this definition had been applied for the current dataset, ~73% of the tails had been scored as intact enough (results not shown).

### Prevalence of Tail Damage

The tail lesion prevalence in the current data is somewhat alarming at first sight, with only about half of the tails being scored as intact. There was, however a clearly observable variation between batches from different farms, which indicates that it is indeed possible to reach a high level of intact tails. The level of tail lesions is higher than the level reported by Valros et al. (4) in a study carried out in 2000 at the same slaughterhouse. It must be noted, however, that the study method, as well as the scoring system, differed largely between these two studies, with the current study using a more detailed protocol, as well as looking more closely at the tails. For example, the majority of the acute wounds in this study were very minor (only a few mm in diameter or length, based on the scorers' experience), which might mean they were not even noted in the less detailed assessment. In the earlier study, it was not possible to physically touch the tails.

It must also be noted that when recordings are done with this level of detail, as compared to the methodology used e.g., for meat inspection (which revealed a total of 0.9% of tail lesions in the current study) the result is much more accurate (6, 29).

Further, when comparing these results to similar studies from countries where the absolute majority of pigs are docked, the amount of non-lesioned tails is in similar ranges. Studies performed in Ireland report between 28 and 72% intact tails (19, 30, 31), in the UK 41% (5), and in Germany 75% (6). Of course, one needs to consider that scoring systems differ between the studies, as well as the detailed scoring protocol in practice. But even so, considering that tail docking has been suggested to have the potential of decreasing tail biting 2-4-fold (32, 33), the situation in this 100% non-docked study population does appear much less worrying. Still, the results do call for further action to reduce the underlying risk factors on farm, especially those occurring during earlier stages of rearing, as the level of fully healed lesions was especially high. The skin of tail wounds caused by docking has been shown to heal within ~4–8 weeks (34). This period might be extended in the case of bitten tails, however, as the bitten tail, as discussed above, might cause a prolonged healing process. Thus, it is not possible to assess, based on this study, if the main problem of biting occurs early in the fattening unit, or before that, in the weaning unit. Anecdotal reports, however, suggest that weaning units are places where the majority of the problems occur in the Finnish pig population (Vugts, personal communication).

We cannot be entirely sure that the week of data sampling was representative for the longer term situation. However, according to the official data on meat inspection outcomes in Finland, the annual tail biting level in all Finnish abattoirs was 1.0% in 2019 (35), which corresponds well with the level found in meat inspection during the week of data collection (0.9%). Further, the percentage of abscesses in the official data from 2019 was 3.0 %, which is also close to what was seen in the current data (e.g., 2.3 % for intact tails), and indicates that there is no reason to assume that this specific week is not at least reasonably representative. As producers were not informed of the study before the data collection began, we can exclude the risk that they might have selected the pigs sent for slaughter during this period.

## Conclusions

These results show that it is possible to measure the level of intact enough tails, as well as different types of lesions during slaughter as a potential estimate of on-farm welfare. By recording different types of lesions, it could be possible to tailor specific advisory measures for farms with different types of problems, thus improving on-farm pig welfare. We suggest developing an official recording system for tail lesions as a part of the official meat inspection, which could help estimate on-farm welfare. A system for scoring tail lesions in undocked pigs should utilize a combination of scoring of the lesion and measuring the tail length. These results have implications for developing automatic recording systems, as both tail lesions and length could be recorded by camera-based systems. It might also be possible to develop systems where only a proportion of the tails are assessed, while still providing reliable benchmark.

As bite marks or bruises on an otherwise intact tail was not a concern for meat hygiene, we suggest the definition of an intact enough tail could allow the inclusion of tails with these mild changes. Meat inspection findings in carcasses with tails scored as healed, but with no fresh lesions, and with more than 75% of the average intact length remaining were rather similar to those of fully intact tails. Based on these findings we suggest that a tail of this length, and with no visible fresh lesions could also be considered intact enough.

## Data Availability Statement

The raw data supporting the conclusions of this article will be made available by the authors, without undue reservation.

## Author Contributions

AV performed the data analysis and HN was responsible for the pathological examinations. All authors contributed to design, data collection, interpretation of the results and preparation of the manuscript. All authors read and approved the final manuscript.

## Conflict of Interest

EV and JV are employed by the company where the study took place, and the study was initiated by the company. An agreement was made prior to the study that the University of Helsinki is responsible for data analysis and scientific publication. The scoring system was developed as a collaboration between the University of Helsinki and the company. The authors thus declare that the company involvement does not cause any bias in the study design, data collection, interpretation of results nor in the preparation of the manuscript. The remaining authors declare that the research was conducted in the absence of any commercial or financial relationships that could be construed as a potential conflict of interest.
